# Pharmacological TLR4 Inhibition Protects against Acute and Chronic Fat-Induced Insulin Resistance in Rats

**DOI:** 10.1371/journal.pone.0132575

**Published:** 2015-07-21

**Authors:** Ning Zhang, Hanyu Liang, Robert V. Farese, Ji Li, Nicolas Musi, Sophie E. Hussey

**Affiliations:** 1 Barshop Institute for Longevity and Aging Studies, The University of Texas Health Science Center at San Antonio, San Antonio, TX, 78229, United States of America; 2 Department of Medicine, University of Texas Health Science Center at San Antonio, San Antonio, TX, 78229, United States of America; 3 The Geriatric Research Education and Clinical Center, South Texas Veterans Health Care System, San Antonio, TX, 78229, United States of America; 4 Department of Internal Medicine, The University of South Florida, Tampa, FL 33620, United States of America; 5 James A Hayley Veterans Medical Center, Tampa, FL, 33612, United States of America; University of Bremen, GERMANY

## Abstract

**Aims:**

To evaluate whether pharmacological TLR4 inhibition protects against acute and chronic fat-induced insulin resistance in rats.

**Materials and Methods:**

For the acute experiment, rats received a TLR4 inhibitor [TAK-242 or E5564 (2x5 mg/kg i.v. bolus)] or vehicle, and an 8-h Intralipid (20%, 8.5 mg/kg/min) or saline infusion, followed by a two-step hyperinsulinemic-euglycemic clamp. For the chronic experiment, rats were subcutaneously implanted with a slow-release pellet of TAK-242 (1.5 mg/d) or placebo. Rats then received a high fat diet (HFD) or a low fat control diet (LFD) for 10 weeks, followed by a two-step insulin clamp.

**Results:**

Acute experiment; the lipid-induced reduction (18%) in insulin-stimulated glucose disposal (Rd) was attenuated by TAK-242 and E5564 (the effect of E5564 was more robust), suggesting improved peripheral insulin action. Insulin was able to suppress hepatic glucose production (HGP) in saline- but not lipid-treated rats. TAK-242, but not E5564, partially restored this effect, suggesting improved HGP. Chronic experiment; insulin-stimulated Rd was reduced ~30% by the HFD, but completely restored by TAK-242. Insulin could not suppress HGP in rats fed a HFD and TAK-242 had no effect on HGP.

**Conclusions:**

Pharmacological TLR4 inhibition provides partial protection against acute and chronic fat-induced insulin resistance *in vivo*.

## Introduction

Through its negative impact on tissues involved in metabolic homeostasis, obesity plays a key role in the development of insulin resistance. Insulin resistance is one of the earliest and most significant abnormalities in the pathogenesis of type 2 diabetes mellitus (T2DM). Alongside efforts to prevent obesity through diet and exercise promotion, there is an urgent need to develop effective therapies to protect against insulin resistance.

Over the past decade, a role for inflammation in the pathogenesis of obesity-mediated insulin resistance increasingly has been recognized. Toll-like receptor (TLR)-4 is a member of the TLR family of pattern recognition receptors, which modulates immune responses by activating inflammatory pathways. Following ligand binding, TLR4 and its co-receptors, CD14 and MD-2, interact with adaptor proteins that facilitate downstream signaling through the IĸB kinase (IKK)-nuclear factor (NF)ĸB complex and the mitogen activated kinase (MAPK) pathways. Several lines of evidence suggest that these inflammatory pathways negatively affect insulin receptor signaling [1]. The chronic nature of obesity produces a tonic low-grade activation of TLR4 that impairs insulin action over time. In rodents and humans with obesity and insulin resistance, elevated expression and downstream signaling of TLR4 has been demonstrated in insulin-target tissues (muscle, liver and adipose tissue) [2–4] and immune cells (monocytes and macrophages) [5, 6]. Although the mechanism(s) for elevated TLR4 signaling are not entirely understood, likely culprits include the classical TLR4 ligand, lipopolysaccharide (LPS) [7, 8] and saturated free fatty acids (FFA) [3, 9, 10], which also function as TLR4 agonists [11]. Both LPS [5, 8, 12] and saturated FFA [13] are elevated in plasma of individuals with obesity and T2DM, in association with the severity of insulin resistance. In line with these observations, experimental elevation (via systemic infusion) of circulating LPS [14] and FFA [15, 16] rapidly induces insulin resistance in normal glucose-tolerant subjects.

Inhibition of TLR4 protects against the detrimental effect of LPS and saturated FFA on inflammation and insulin action in muscle cells *in vitro* [8–10]. Moreover, most, albeit not all [17] studies in genetically modified mice have shown that disrupted TLR4 function protects against acute and chronic fat-induced impairments in insulin action *in vivo* [3, 4, 9, 18]. In this regard, pharmacological inhibitors of TLR4 might be useful therapeutics in the treatment of insulin resistance and T2DM. TAK-242 (resatorvid), a cyclohexene derivative, is a small-molecule inhibitor of TLR4 signaling, which binds selectively to Cys^747^ in the TIR domain of TLR4 [19] and subsequently disrupts the ability of TLR4 to associate with toll-interleukin 1 receptor (TIR) domain containing adaptor protein [20]. Another widely studied TLR4 inhibitor, E5564 (eritoran tetrasodium), competitively and selectively binds to TLR4-MD2 and inhibits an agonist from initiating an inflammatory response [21]. Both TAK-242 [22, 23] and E5564 [24, 25] have been characterized as novel anti-sepsis agents capable of inhibiting inflammatory mediator production; the compounds block NFĸB activation and cytokine production following LPS stimulation *in vitro* and *in vivo*, both in rodents and humans with experimental endotoxemia. Although TAK-242 [26] and E5564 [27] were ineffective in significantly reducing mortality in severely septic patients, TLR4 inhibitors may be beneficial for low-grade inflammatory diseases characterized by insulin resistance. Indeed, we recently characterized TAK-242 as an efficacious inhibitor of inflammation (NFĸB/MAPK pathways) and insulin resistance in muscle cells *in vitro* [8, 28]. In the present study, we sought to examine the effect of TAK-242 and E5564 on insulin action *in vivo* by utilizing two well-established models of fat-induced insulin resistance (acute lipid infusion and chronic high fat feeding). We hypothesized that pharmacological TLR4 inhibition would protect against hepatic and peripheral insulin resistance in rats challenged with lipid infusion or high fat feeding.

## Materials and Methods

### Animals

Male Sprague-Dawley rats (6 weeks old) and male Long Evans rats (9 weeks old) were obtained from Charles River. Rats were provided *ad libitum* access to food and water and were housed in 12-h light-dark cycles. The Office of the Institutional Animal Care Program at The University of Texas Health Science Center at San Antonio approved all procedures performed in this study.

### Acute lipid infusion study intervention

Following a 7-day acclimatization period, Sprague-Dawley rats were anesthetized and catheters were implanted into the left common carotid artery and the right jugular vein as previously described [29]. After 4-days of recovery, fasted (~12 h), conscious, unrestrained rats were randomized to receive 2 x bolus TAK-242 (5 mg.kg^−1^, Chemleader Biomedical Co. ltd, Shanghai, China), E5564 (5 mg.kg^−1,^ Eisai Pharmaceuticals, Andover, MA) or vehicle through the indwelling arterial catheter. Intralipid 20% (8.5 mg.kg^−1^min^−1^) or saline were infused for 8-h. Insulin sensitivity was measured by a two-step (designated step I and II) hyperinsulinemic-euglycemic clamp. The insulin clamp started with a priming injection (10 μCi/0.2 ml) and constant infusion (0.1 μCi.min^−1^) of d-[3-^3^H]-glucose (Perkin Elmer, Waltham, MA). After 60-min of tracer equilibration, insulin (Novo Nordisk, Princeton, NJ) was infused at a low dose rate of 0.4 mU.m^2^.min^−1^ into the jugular vein (step I: 0–120-min) to measure whole body insulin sensitivity, particularly the affect of hepatic insulin sensitivity [suppression of hepatic glucose production (HGP)]. The insulin infusion rate was increased to 4 mU.m^2^.min^−1^ (step II: 120–240 min) to primarily measure peripheral (muscle) insulin sensitivity since hepatic glucose production was suppressed totally. Somatostatin (Sigma Aldrich, St Louis, MO) was infused (3 μg.kg^−1^.min^−1^) during the clamp to suppress endogenous insulin release and 20% dextrose (Sigma) was infused at a various rate to maintain constant glucose concentrations. Achievement of steady-state conditions was confirmed by ensuring glucose levels were maintained constant for a minimum of 30 min (CV <5%). Blood glucose was measured every 10 min using a GM300 glucose meter (Bionime, San Diego, CA). Blood samples were obtained at *t* = −60, -20, −10, 0, 80, 90, 100, 110, 120, 200, 210, 220, 230 and 240 min. All samples were immediately centrifuged and plasma was stored at −80°C for subsequent analysis. Plasma d-[3-^3^H]-glucose specific activity was measured using liquid scintillation counting. The mean (steady state) concentrations/rates from -20 to 0 min (basal), 90 to 120 min (step I) and 210 to 240 min (step II) were used for calculations. Under steady-state conditions, the rate of whole body glucose disappearance (Rd) equals the rate of whole body glucose appearance and was calculated by dividing the infusion rate of d-[3-^3^H]-glucose (dpm) by the steady state of d-[3-^3^H]-glucose specific activity. The rate of HGP was calculated by subtracting the exogenous glucose infusion rate from whole body glucose appearance.[30].

### Chronic diet-induced obesity study intervention

Following a 1 week acclimatization period, Long Evans rats were randomized to receive TAK-242 or placebo via the Matrix-Driven Delivery (MDD) pellet system (Innovative Research of America, Saratosa, FL). MDD pellets were implanted subcutaneously between the scapulae on the upper back of the rat. Pellets (70-day release) contained 105 mg of TAK-242, designed to administer 1.5 mg/day. Placebo pellets contained no active ingredient. Immediately following pellet implantation, rats were randomly assigned to a high fat diet (HFD; 60% calories from fat, TD 06414) or a low fat control diet (LFD; 10% calories from fat, TD 08806) (Harlan Laboratories, Madison, WI), provided *ad libitum*. During the study, body weight and food consumption were measured weekly. Following ~70 days of experimental feeding, rats were anesthetized and catheters were implanted into the left common carotid artery and the right jugular vein as previously described [29]. After 4-days of recovery, a two-step hyperinsulinemic-euglycemic clamp was conducted as described above, with the following modifications to the insulin dose to account for the rats higher body weight; Insulin was infused at a low-dose rate of 2 mU.m^2^.min^−1^ (step I: 0–120 min) and a higher-dose rate of 4.0 mU/m^2^.min (step II: 120–240 min).

### Statistical analysis

All data are represented as the mean ± SE. Data were evaluated for statistical significance by 1-way ANOVA with Turkey multiple comparison tests or 2-way ANOVA with Bonferroni multiple comparison tests (GraphPad Software, San Diego, CA).

## Results

### Acute Lipid Infusion Study

#### Glucose concentrations and GIRs during the hyperinsulinemic-euglycemic clamp

**Fig 1:**
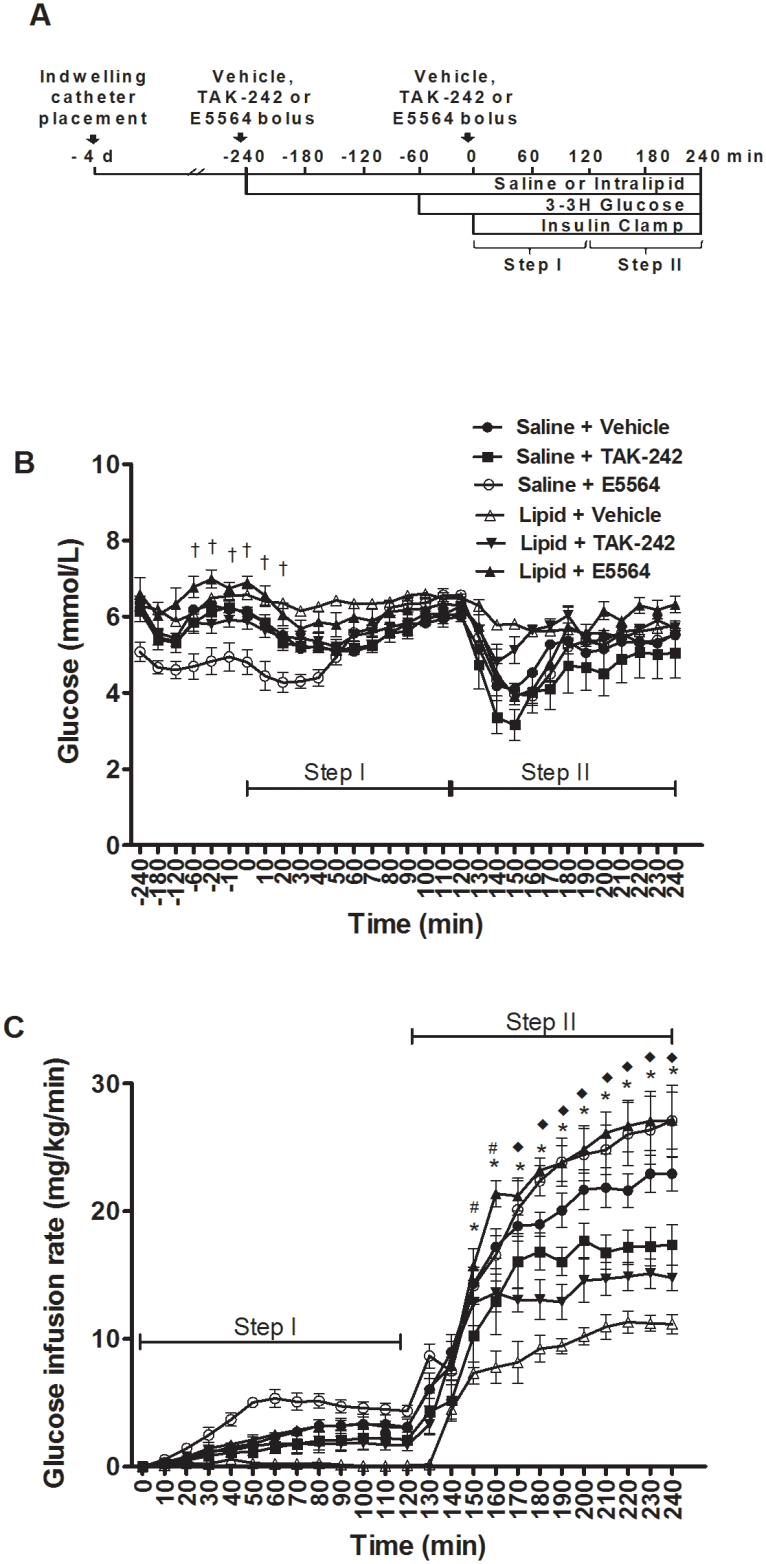
Protocol for the acute lipid infusion study intervention (A). Blood glucose concentrations (B) and glucose infusion rates (C) in rats infused with saline or lipid and administered vehicle, TAK-242 or E5564. Values are means ± SEM, *n* = 6–9 rats/group. *Saline+Vehicle vs. Lipid+Vehicle; ^#^Lipid+Vehicle vs. Lipid+TAK-242; ^◆^Lipid+vehicle vs. Lipid+E5564, ^†^Saline+Vehicle vs. Saline+E5564 (p<0.05).

The protocol for the acute lipid infusion study is shown in Fig 1A. Similar fasting glucose levels were observed in all groups (Fig 1B). No differences were observed in mean glucose levels during the low-dose steady state insulin infusion period (step I) or the high dose steady state insulin infusion period (step II) (Fig 1B). As expected, the GIR required to maintain euglycemia increased with the higher insulin dose infusion (Step I vs. Step II, Fig 1C). The GIR was significantly lower in the Lipid+Vehicle group compared to the following groups; Saline+Vehicle (at t = 150 min through t = 240 min); Lipid+E5564 (at t = 170 min through t = 240 min) and; Lipid+TAK-242 (at t = 150/160 min) (p<0.05, Fig 1C).

#### TAK-242 and E5564 ameliorate lipid-induced reductions in peripheral glucose disposal

**Fig 2:**
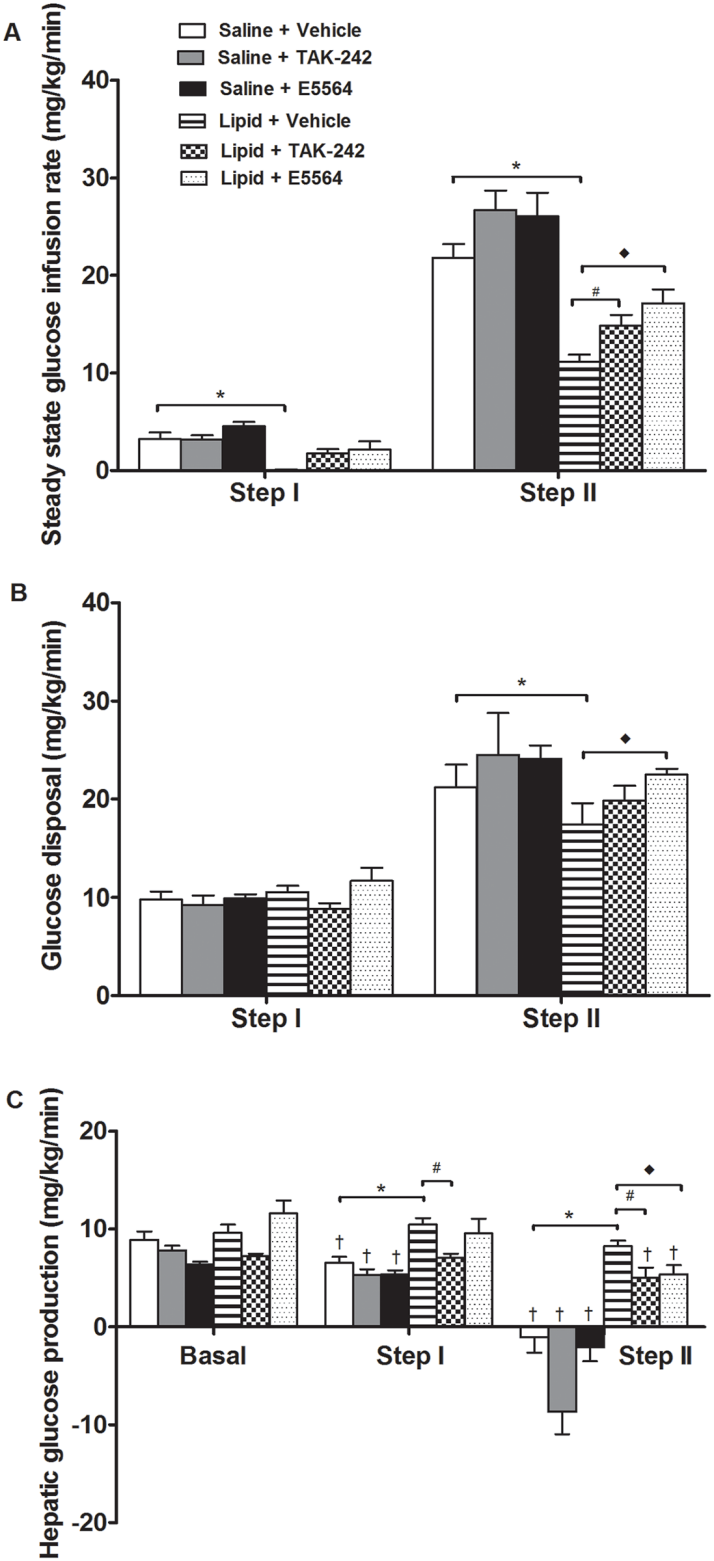
Steady state glucose infusion rates (A), glucose disposal (B) and hepatic glucose production (C) in rats infused with saline or lipid and administered vehicle, TAK-242 or E5564. Values are means ± sem, *n* = 6–9 rats/group. *Saline+Vehicle vs. Lipid+Vehicle; ^#^Lipid+Vehicle vs. Lipid+TAK-242; ^◆^Lipid+Vehicle vs. Lipid+E5564, ^†^suppression from basal (p<0.05). *Note*: *negative HGP values are an artifact and suggest complete suppression*.

The steady-state GIR required to maintain euglycemia in rats infused with lipid was ~30% (step I) and ~50% (step II) of the GIR in rats infused with saline (p<0.05, Fig 2A). TAK-242 and E5564 increased the GIR in lipid-infused rats during step-II of the clamp, with the effect of E5564 being more robust (p<0.05, Fig 2A). Accordingly, lipid caused a small reduction (~18%) in insulin-stimulated glucose disposal (Rd) during step II of the clamp (p<0.05, Fig 2B). This effect was partially attenuated by E5564 (p<0.05, Fig 2B). The lipid infusion also exerted a lesser effect on the Rd during step II in rats administered TAK-242. However, this effect did not reach statistical significance, probably due to the modest effect of lipid on insulin-stimulated Rd (Fig 2B). Taken together, our findings suggest that TAK-242 and E5564 improve peripheral (muscle) insulin action in lipid-treated rats, with the effect of E5564 being more robust.

#### TAK-242 and E5564 ameliorate lipid-induced insulin resistance in the liver

Basal HGP was similar in all groups (Fig 2C). In saline-infused rats, insulin caused a dose-dependent suppression of HGP; a ~27% reduction during step I and complete suppression during step II of the clamp (p<0.05, Fig 2C). Notably, HGP during step II was estimated to be negative in rats administered saline, probably reflecting limitations of the fixed-rate infusion technique in situations whereby there is relatively high glucose turnover [31]. Such negative values are an artifact and have conventionally been taken to indicate complete suppression of HGP [31]. During step I and step II of the clamp, the suppression of HGP was lower in lipid-treated rats, compared to saline (p<0.05, Fig 2C). TAK-242 restored the ability of insulin to suppress HGP in lipid treated rats during step I of the clamp (p<0.05). However, the effects of E5564 did not reach statistical significance (Fig 2C). Nonetheless, the ability of insulin to suppress HGP was markedly and equally enhanced by TAK-242 and E5564 during step II (p<0.05, Fig 2C). Collectively, these results indicate that TAK-242 treatment leads to robust effects on hepatic insulin sensitivity, with the effects of E5564 being more modest.

### Chronic Diet-induced Obesity Study

#### Body weight and energy intake

**Fig 3:**
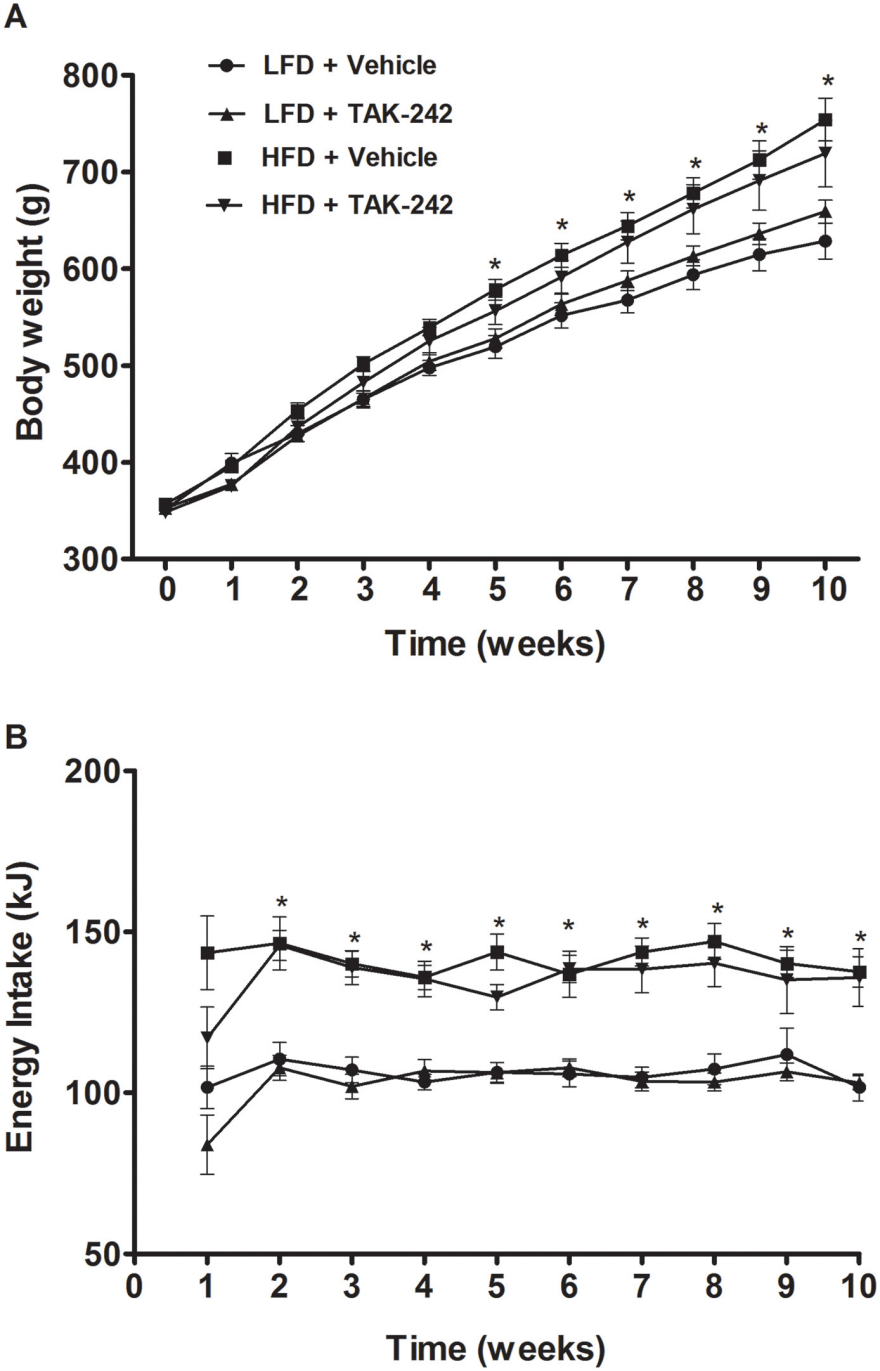
Body weights (A) and energy intake (B) of rats fed a HFD or a LFD and administered TAK-242 or vehicle for 10 wk. Values are means ± sem, *n* = 8–10 rats/group. *LFD+Vehicle vs. HFD+Vehicle (p<0.05).

Starting from 5-weeks into the study, rats fed a HFD had significantly greater body weight than rats fed a LFD diet (2A, p<0.05). Rats fed a HFD diet continued to gain more body weight such that by 10-weeks into the study they were ~20% heavier than rats fed a LFD (Fig 3A, p<0.05); TAK-242 had no effect on body weight in rats fed either a LFD (591±24 g and 614±11 g in vehicle and TAK-242 treated rats respectively) or a HFD (731±23 g and 698±35 g in vehicle and TAK-242 treated rats respectively) when assessed 10-weeks into the study (Fig 3A). Starting from 2-weeks into the study, energy intake was relatively consistent throughout the 10-week intervention in all groups (Fig 3B). On average, rats fed a HFD had ~30% greater energy intake compared to rats fed a LFD (p<0.05, Fig 3B). TAK-242 had no significant effect on energy intake in rats fed either a LFD (106±1 and 106±4 kcal in vehicle and TAK-242 treated rats respectively) or a HFD (141±1 and 135±2 kcal in vehicle and TAK-242 treated rats respectively) (Fig 3B).

#### Glucose concentrations and GIRs

**Fig 4:**
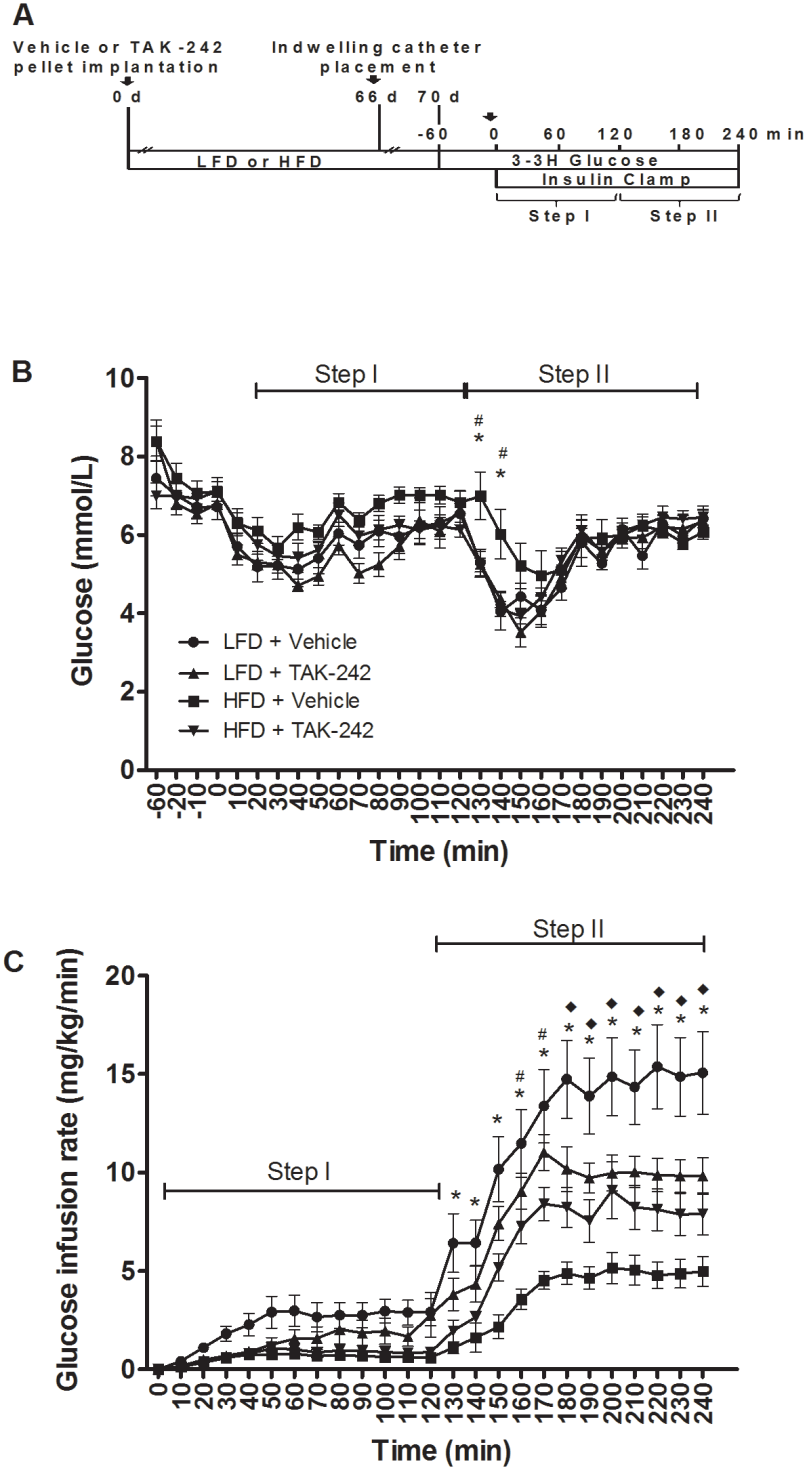
Protocol for the chronic diet-induced obesity study (A). Blood glucose concentrations (B) and glucose infusion rates (C) in rats fed a HFD or a LFD and administered TAK-242 or vehicle for 10 wk. Values are means ± sem, *n* = 8–10 rats/group. *LFD+Vehicle vs. HFD+Vehicle; ^#^HFD+Vehicle vs. HFD+TAK-242, ^◆^LFD+Vehicle vs. LFD+TAK-242 (p<0.05).

The protocol for the diet-induced obesity study is shown in Fig 4A. Fasting glucose levels were similar in all groups (Fig 4B). No differences were observed in mean glucose levels during the low-dose steady state insulin infusion period (step I;) or the high dose steady state insulin infusion period (step II) (Fig 4B). As expected, the GIR required to maintain euglycemia increased with the high insulin dose (Step I vs. Step II, Fig 4C). The GIR was significantly lower in the HFD+Vehicle group compared to the following groups; LFD+Vehicle (at t = 130 min through t = 240 min); HFD+TAK-242 (at t = 170 min through t = 240 min) and; LFD+TAK-242 (at t = 160/170 min).

#### TAK-242 ameliorates HFD-induced reductions in peripheral glucose disposal

**Fig 5:**
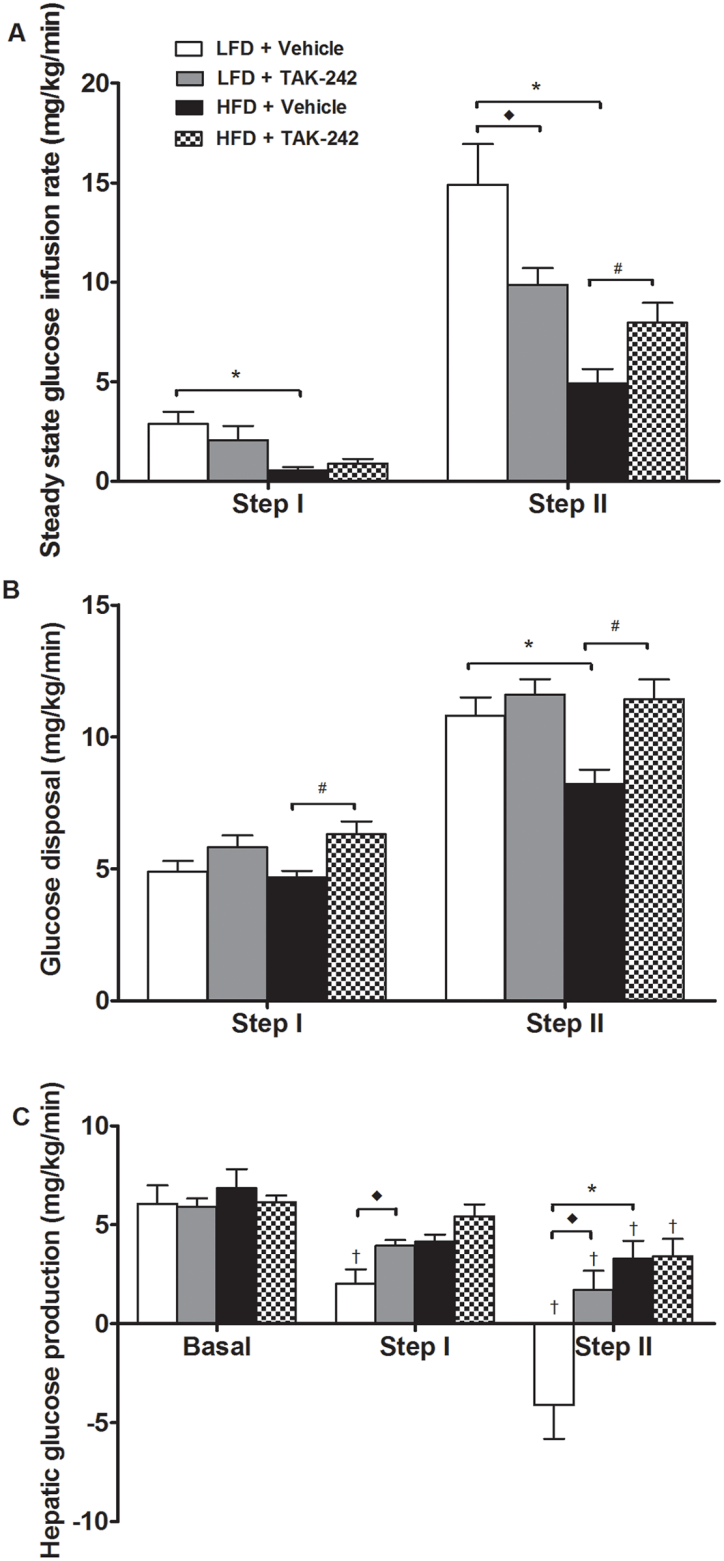
Steady state glucose infusion rates (A), glucose disposal (B) and hepatic glucose production (C) in rats fed a HFD or a LFD and administered TAK-242 or vehicle for 10 wk. Values are means ± sem, *n* = 8–10 rats/group. *LFD+Vehicle vs. HFD+Vehicle, ^◆^LFD+Vehicle vs. LFD+TAK-242, ^†^suppression from basal (p<0.05). *Note*: *negative HGP values are an artifact and suggest complete suppression*.

The steady-state GIR required to maintain euglycemia in rats administered the HFD was ~20% (step I) and ~30% (step II) of the GIR in rats administered the LFD (Fig 5A, p<0.05). During step II, administration of TAK-242 partially ameliorated the reduction in the GIR in rats fed a HFD; the GIR was significantly greater in the HFD+TAK-242 group compared to the HFD+Vehicle group (p<0.05, Fig 5A). Consistent with these findings, the HFD caused a reduction (~30%) in insulin-stimulated Rd during step II of the clamp (p<0.05) and this effect was completely restored by TAK-242 (p<0.05, Fig 5B). Together, these findings imply that TAK-242 offers protection against HFD-induced peripheral insulin resistance. Surprisingly, in rats administered the LFD, TAK-242 reduced the GIR during step II of the clamp (p<0.05, Fig 5A) but had no effect on insulin-stimulated Rd (Fig 5B).

#### TAK-242 has no effect on HFD-induced insulin resistance in the liver

Basal HGP was similar in all groups (Fig 5C). As anticipated, insulin caused a dose-dependent suppression of HGP rats fed a LFD; ~ 67% reduction during step I and complete suppression during step II of the clamp, when compared to basal HGP (p<0.05, Fig 5C). The ability of insulin to suppress HGP was impaired in rats fed a HFD, inducing a smaller ~33% reduction in HGP during step I of the clamp and offering no further suppression during step II of the clamp (p<0.05, Fig 5C). Notably, compared to vehicle, TAK-242 did not affect the ability of insulin to suppress HGP during step I or II of the clamp in rats fed a HFD (Fig 5C). This finding suggests that TAK-242 was unable to restore hepatic insulin sensitivity. Surprisingly, in rats fed a LFD, TAK-242 increased HGP during step I of the clamp when compared to vehicle (p<0.05, Fig 5C). Moreover, during step II of the clamp, the complete suppression in HGP observed in vehicle treated rats was absent in TAK-242 treated rats fed a LFD (Fig 5C).

## Discussion

The present study is the first to evaluate the effect of pharmacological TLR4 inhibition on acute and chronic fat-induced insulin resistance *in vivo*. In general, studies have shown a positive effect of genetic TLR4 disruption (deletion or loss-of-function) on glucose metabolism [3, 4, 9, 32]. However, some controversy exists as to which insulin–sensitive tissues are responsible for this systemic effect. While some studies report enhanced insulin action in adipose tissue, muscle and liver [3, 4, 9], others challenge improvements in muscle [32] and liver [17]. Using two well-established models of fat-induced insulin resistance (acute lipid infusion and chronic HFD administration), we evaluated the effects of pharmacological TLR4 inhibition on peripheral and hepatic insulin action. We hereby show that TLR4 inhibition had an efficacious capacity to restore insulin action in rats infused with lipid; both TAK-242 and E5564 partially reversed lipid-induced reductions in insulin-stimulated Rd, and improved the ability of insulin to suppress HGP, indicating improved insulin action in both muscle and liver. Notably, the ability of E5564 to restore peripheral glucose disposal was more robust than that of TAK-242. On the contrary TAK-242 was more efficacious than E5564 in enhancing insulin’s ability to suppress HGP. Some important differences between TAK-242 and E5564 exist which could explain their divergent efficacy. The mechanism of action likely plays a key role; E5564 binds to the TLR4/MD-2 complex, thereby inhibiting TLR4 activation [21], whereas TAK-242 binds to Cys747 in the intracellular domain of TLR4, subsequently inhibiting the protein’s functionality [19, 20]. Differences in the compounds molecular weight and lipid solubility could also be important; E5564 has a large molecular weight (1401.6) and low liposolubility, whereas TAK-242 has a low molecular weight (360.1) and high liposolubility. These divergent physical and chemical properties could affect the bioavailability, solubility, absorption capacity and/or distribution in tissues.

We further studied TAK-242 in a model of HFD-induced insulin resistance. Chronic TAK-242 administration had a clear effect on insulin action in the muscle; completely restoring insulin-stimulated Rd in rats challenged with a HFD. Surprisingly, TAK-242 had no effect on insulin’s ability to suppress HGP, suggesting no beneficial effect on insulin action in the liver. In this regard, the lipid infusion model of insulin resistance was not entirely predictive of findings in the HFD model. This could be a result of differences in the strain, weight or age of rats studied under the different models. However, another explanation involves the more complex nature of the prolonged HFD intervention, which likely introduced several factors that contribute to the livers production of glucose, independent of TLR4. One putative mechanism involves the accumulation of intracellular diacyglycerols (DAGs) as a result of excess lipid delivery to the liver and/or reductions in fatty acid oxidation. This increase in hepatic DAG content leads to activation of PKCε which, in turn, inhibits insulin-stimulated insulin receptor kinase phosphorylation of IRS proteins and impairs insulin signaling at the level of IRS-2, [1, 17]. The apparent inability of TAK-242 inhibition to restore HFD-induced insulin action in the liver is in disagreement with some studies in mice with genetic TLR4 disruption [3, 4, 32]. Fundamental differences between pharmacological and genetic inhibition of TLR4 may explain the findings, such that ‘off-target effects’ are possible in either model. It is also important to note that previous studies have typically utilized indirect measures of insulin action such as glucose and insulin tolerance tests, and/or markers of insulin signaling [3, 32], or the one-step hyperinsulinemic-euglycemic clamp [4, 17], which typically reflects insulin action in skeletal muscle [33]. The present study utilized the two-step hyperinsulinemic-euglycemic clamp to obtain information on insulin action at the lower and upper end of the physiological range of insulin concentration, thereby providing a comprehensive estimate of insulin effects in the liver and muscle. The finding that TAK-242 reduced the GIR during step II of the clamp in rats administered the LFD was indeed surprising. The lower GIR required to maintain euglycemia was caused by the increase in hepatic glucose production.

While most previous studies support the paradigm that genetic disruption of TLR4 protects against whole-body insulin resistance [3, 4, 9, 18], the effect of TLR4 disruption on body weight and adiposity in response to a HFD is less clear. In TLR4 knockout mice, an obese phenotype [3] as well as no change in body weight [34] have been described. Moreover, in mice with a loss of function in TLR4, both increased [32, 35] and decreased [4, 36, 37] weight gain has been reported. The mechanism(s) for the apparent changes in body weight and adiposity are also controversial, with some studies describing alterations in food intake [3, 32] and others reporting changes in energy expenditure [4, 36, 37]. Explanations for the discrepant results are not clear but may include differences in the nature of the genetic alteration of TLR4, weight, gender or strain of the mouse used and/or differences in the composition or duration of the HFD. Since the effects of TLR4 disruption on insulin action may be secondary to observed changes in body weight and/or adiposity, the conflicting findings make it somewhat difficult to assess the direct effects of TLR4 on insulin action. In the present study, TAK-242 treatment had no effect on body weight or energy intake, which suggests that changes in glucose metabolism were a direct consequence of TLR4 inhibition. Although we cannot rule out ‘off-target effects’ of the TLR4 inhibitor *in vivo*, specificity of the inhibitor has been extensively studied and confirmed in vitro [19, 20].

In conclusion, TAK-242 and E5564 partially reversed acute lipid-induced insulin resistance in rats by lowering hepatic glucose production and restoring insulin-stimulated Rd. TAK-242 also enhanced insulin-stimulated Rd in rats challenged with a HFD. Our findings support the paradigm that TLR4 represents a novel target for the treatment of insulin resistance and that TLR4 inhibitors could be useful therapeutics in the management of obesity-related metabolic disease.
